# A systematic review of historical and contemporary evidence of trachoma endemicity in the Pacific Islands

**DOI:** 10.1371/journal.pone.0207393

**Published:** 2018-11-15

**Authors:** Becca L. Handley, Chrissy h. Roberts, Robert Butcher

**Affiliations:** Clinical Research Department, London School of Hygiene & Tropical Medicine, London, United Kingdom; University of California, UNITED STATES

## Abstract

**Introduction:**

Trachoma is endemic in several Pacific Island countries. The aims of this study were to (a) identify future trachoma mapping needs in the Pacific and (b) to examine whether any temporal trends in trachoma prevalence could be ascertained from the historical literature on trachoma in the Pacific Islands.

**Methods:**

Human studies of trachoma and eye care in the Pacific Islands were identified from a systematic search of PubMed, EMbase, Scopus and Web of Science databases. A published quality assessment system for disease prevalence studies was modified to assess studies for quality and transparency.

**Results:**

Few general ophthalmic studies in the Pacific mention trachoma. In targeted studies of trachoma, cases have consistently been identified throughout the Pacific since the early twentieth century. The largest number of studies come from Papua New Guinea and Fiji, whereas some countries have no published data on trachoma. The majority of studies identified were published before the Alliance for the Global Elimination of Trachoma 2020 was convened, so lack the standardisation of population-based mapping which has been implemented in the past decade.

**Conclusions:**

Population-based trachoma prevalence estimates have been recently generated in Papua New Guinea, Solomon Islands, Vanuatu, Kiribati and Fiji. There is insufficient evidence to assess whether there has been temporal change in trachoma prevalence in these countries over the past century. Cases of trachoma have been identified in some countries (for example, Nauru and Samoa) which have no recent population-based mapping data, but may be at risk of trachoma endemcitiy. Deployment of appropriate mapping strategies is warranted to identify whether interventions are required.

## Introduction

Trachoma is a chronic keratoconjunctivitis caused by ocular serovars (A-C) of *Chlamydia trachomatis* (*Ct*). Infection with *Ct* is most common in young children and results in follicular and papillary inflammation. Repeated rounds of infection, inflammation and resolution can lead to conjunctival scarring and may eventually cause eyelashes to turn inward and abrade the globe of the eye. Abrasion from eyelashes can cause opacification of the cornea, the sight-threatening stage of trachoma [1]. Over the past two decades, there has been a concerted global effort to eliminate blinding trachoma as a public health problem by 2020. This is supported by the WHO-led Global Elimination of Trachoma by 2020 (GET2020) Alliance, convened in 1996 [2], and by World Health Assembly resolution 51.11 which was passed in 1998 [3]. An effective package of community-wide control interventions has also been developed and this is known as the ‘SAFE’ strategy. SAFE denotes: **S**urgery to treat those suffering with trachomatous trichiasis (TT), mass drug administration (MDA) with **A**ntibiotics, promotion of **F**acial cleanliness and **E**nvironmental improvement to reduce prevalence and transmission of the causative agent [4]. The WHO Simplified Grading Scheme [5], a standardisable, scalable diagnostic system, has been used in the generation of high-quality, district-level maps of trachoma prevalence. Current recommendations suggest implementation of the SAFE strategy to reduce the population prevalence of TT to <0.1% and the prevalence of trachomatous inflammation–follicular (TF) in 1–9 year olds to <5% [6]. Although perhaps not on course for global elimination by 2020, overall progress towards the goal is good. Much of the research and focus of trachoma control efforts takes place in Africa, where the majority of the global burden of disease persists [7]; however, trachoma studies are lacking in several other regions.

The Pacific region consists of thousands of volcanic islands and coral atolls dispersed over vast seas. Demographically, these islands are mainly populated by individuals descended from one of three genetically distinct indigenous ethnicities: Melanesian, Polynesian and Micronesian [8]. The region faces unique challenges in the provision of eyecare services due to its geography. A 2014 review of visual impairment in the Pacific suggested that 0.5–1.2% of the Pacific Island population were blind [9]. In that study, the leading causes of blindness were listed as cataracts (responsible for 40.6% of blindess), uncorrected refractive error (13.6%), macular degeneration (4.6%), glaucoma (4.2%) and diabetic retinopathy (1.2%). Recent Global Trachoma Mapping Project (GTMP) and International Agency for the Prevention of Blindness (IAPB) surveys have indicated that TF remains widespread throughout the region, but these surveys also suggested that the prevalence of TT and ocular *Ct* infection were low given the corresponding TF prevalences [10–16]. This discrepant clinical presentation presents a problem for policymakers, as they must decide whether to implement costly community-wide treatment strategies in a region where the correlation between clinical signs and risk of sight-threatening disease is unclear. For these reasons, during a 2018 meeting convened by the WHO, local stakeholders endorsed the inclusion of infection testing and more detailed investigation into the presentation of trachoma to determine whether implementation of the SAFE strategy is appropriate [17]. Recent surveys in the Pacific have also highlighted how trachoma prevalence could be unevenly distributed between neighbouring islands or regions of the same nation [10,16]. This is perhaps unsurprising given the geographical isolation of many of the islands, but raises questions regarding whether data from population-based surveys of island chains can be legitimately extrapolated to wider regions or nationally.

This study has systematically reviewed historical and contemporary literature on trachoma in the Western Pacific. Our aim was to provide an overview of the current epidemiological picture with a historical context that may help with the interpretation of the unusual clinical data observed in some surveys. By amalgamating trachoma data collected over the past century, we attempted to investigate temporal trends. Further, by identifying a number of populations for which very little or no data exists, we hoped to highlight underserved regions and nations which may need mapping. To better understand the relative contribution of trachoma to overall blindness rates in the Pacific, we chose to include in our review all studies of visual impairment and blindness that focus on this region.

## Methods

### Scope of the review

The review focuses on states in the Pacific. For the purposes of this review, we focussed on members of the Secretariat of the Pacific Community (SPC) [18]. The current population, political status [19]and trachoma-endemicity status (according to the Global Health Observatory [20]) of each state included in this review are displayed in Table 1. The USA (except Hawaii, due to its large Polynesian population), France, New Zealand and Australia are members of the SPC but were excluded as they do not share sociodemographic, economic and/or geographic characteristics with the target states. Both those classified as endemic and non-endemic for trachoma were included in the search terms to improve our understanding of the spatial and temporal distribution of trachoma throughout the region.

**Table 1:** Selected nations for review.

Region	Country/territory	Population (based on most recent census)	Political Status *	Trachoma endemicity status in 2017 **
Melanesia	Papua New Guinea	7,275,324	Independent nation	Known to require interventions
	Fiji	884,887	Independent nation	Known to require interventions
	Solomon Islands	515,870	Independent nation	Known to require interventions
	Vanuatu	272,459	Independent nation	Known to require interventions
	New Caledonia	269,000	Territorial collectivity of France	Non-endemic
Micronesia	Guam	159,358	Organised, unincorporated territory of the US	-
	Kiribati	110,136	Independent nation	Known to require interventions
	Federated States of Micronesia	102,624	Federal republic in free association with the US	Non-endemic
	Northern Mariana Islands	55,883	Commonwealth in political union with the US	-
	Marshall Islands	53,158	Presidential republic in free association with the US	Non-endemic
	Republic of Palau	17,661	Presidential republic in free association with the US	Non-endemic
	Nauru	10,084	Independant nation	Status uncertain
Polynesia	French Polynesia	275,918	Overseas collectivity of France	Non-endemic
	Samoa	195,979	Independent nation	Non-endemic
	Tonga	100,651	Independent nation	Non-endemic
	American Samoa	55,519	Unincorporated and unorganised territory of the US;	Non-endemic
	Cook Islands	17,794	Self-governing in free association with New Zealand	Non-endemic
	Wallis and Furtuna	13 484	Overseas collectivity of France	Non-endemic
	Tuvalu	10,837	Independent nation	Non-endemic
	Niue	1607	Self-governing in free association with New Zealand	Non-endemic
	Tokelau	1499	Self-administering territory of New Zealand	-
	Pitcairn Islands	66	Overseas territory of the UK	-

* Political status taken from the US Central Intelligence Agency World Fact Book [19]

** Endemicity classification taken from the Global Health Observatory [20]. Places with status listed on the Global Health Observatory were shown with a hyphen (-)

### Search strategy

The PRISMA guidelines were used to develop a search strategy for this review (S1 Table) [21]. Literature searches were conducted in parallel by two independent investigators. Final decisions on article inclusion were made following discussion between the two investigators. Searches of publicly-available information were conducted using PubMed, EMbase, Scopus and Web of Science. The following search terms were used:

- Trachoma OR (ocular AND trachomatis) OR Blindness OR conjunctivitis OR “eye disease” OR “visual impairment” OR “neglected tropical disease”
- AND Pacific OR Polynesia OR Micronesia OR Melanesia OR “American Samoa” OR “Cook Islands” OR “Federated States of Micronesia” OR Fiji OR “French Polynesia” OR Guam OR Kiribati OR “Marshall Islands” OR Nauru OR “New Caledonia” OR Niue OR “Northern Mariana Islands” OR Palau OR “Papua New Guinea” OR “Pitcairn Islands” OR Samoa OR “Solomon Islands” OR Tokelau OR Tonga OR Tuvalu OR Vanuatu OR “Wallis and Futuna”

Only human studies were included in this review. All studies published before 1^st^ July 2018 were eligible for inclusion. Studies published prior to 1996 (when the WHO convened the alliance for the GET2020 campaign) will be referred to as ‘historical’ studies; those published after will be referred to as ‘contemporary’ studies. The study includes articles not written in English. Reference lists of identified articles were screened for further publications of interest. Manual searches to identify any other relevant material for inclusion were conducted.

### Data extraction and quality assessment

Criteria for assessing the quality of shortlisted studies which describe trachoma prevalence were set out prior to data extraction. These criteria, described in S2 Table, were based on published objective criteria for assessing population prevalence studies for systematic reviews [22]. Relevant information on clinical epidemiology of trachoma was extracted from shortlisted articles. The synthesis of data was primarily descriptive; meta-analysis of risk factors was beyond the scope of this review.

## Results

**Fig 1:**
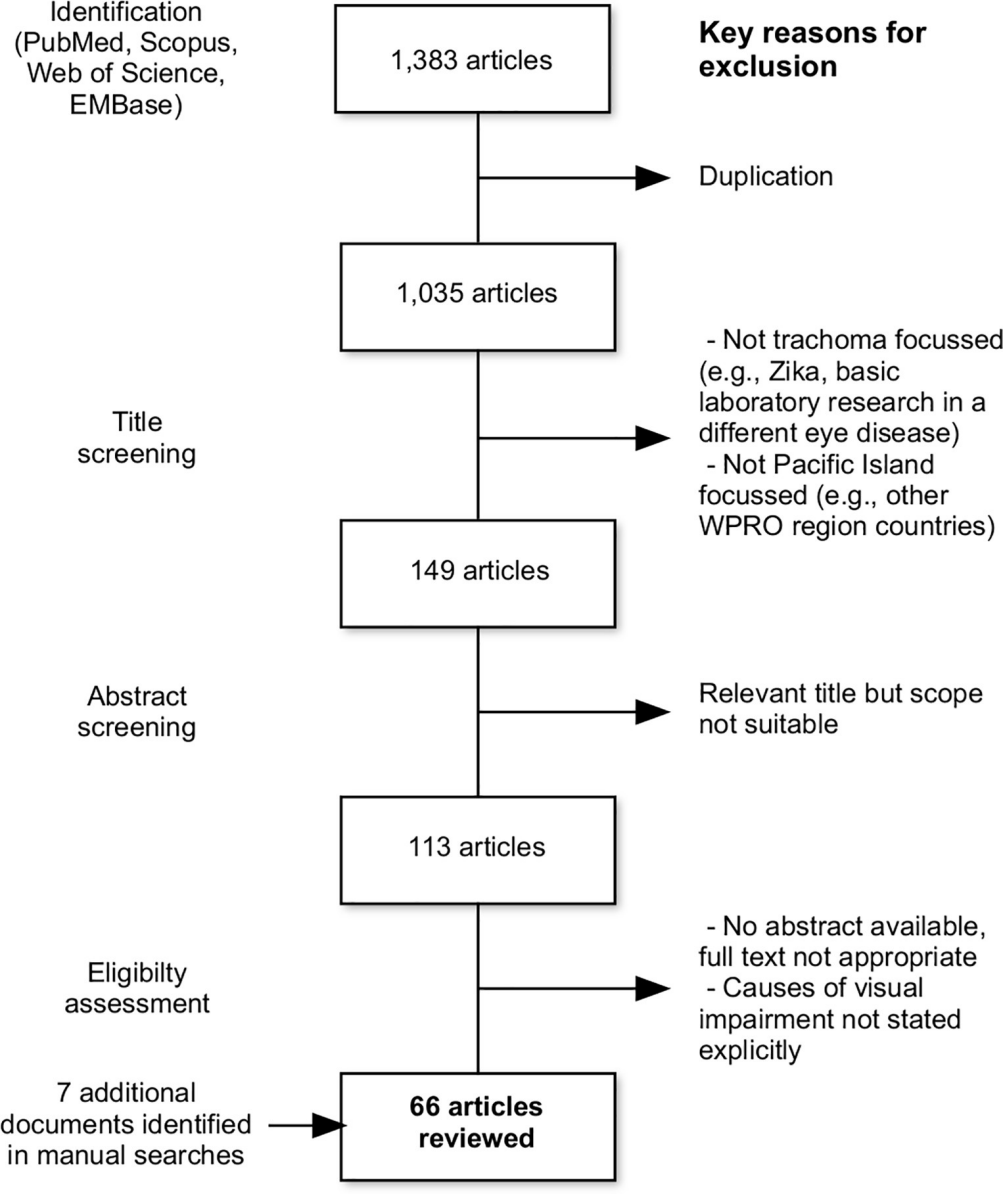
Flowchart of articles included in this systematic review and key reasons for exclusion at each stage.

**Fig 2:**
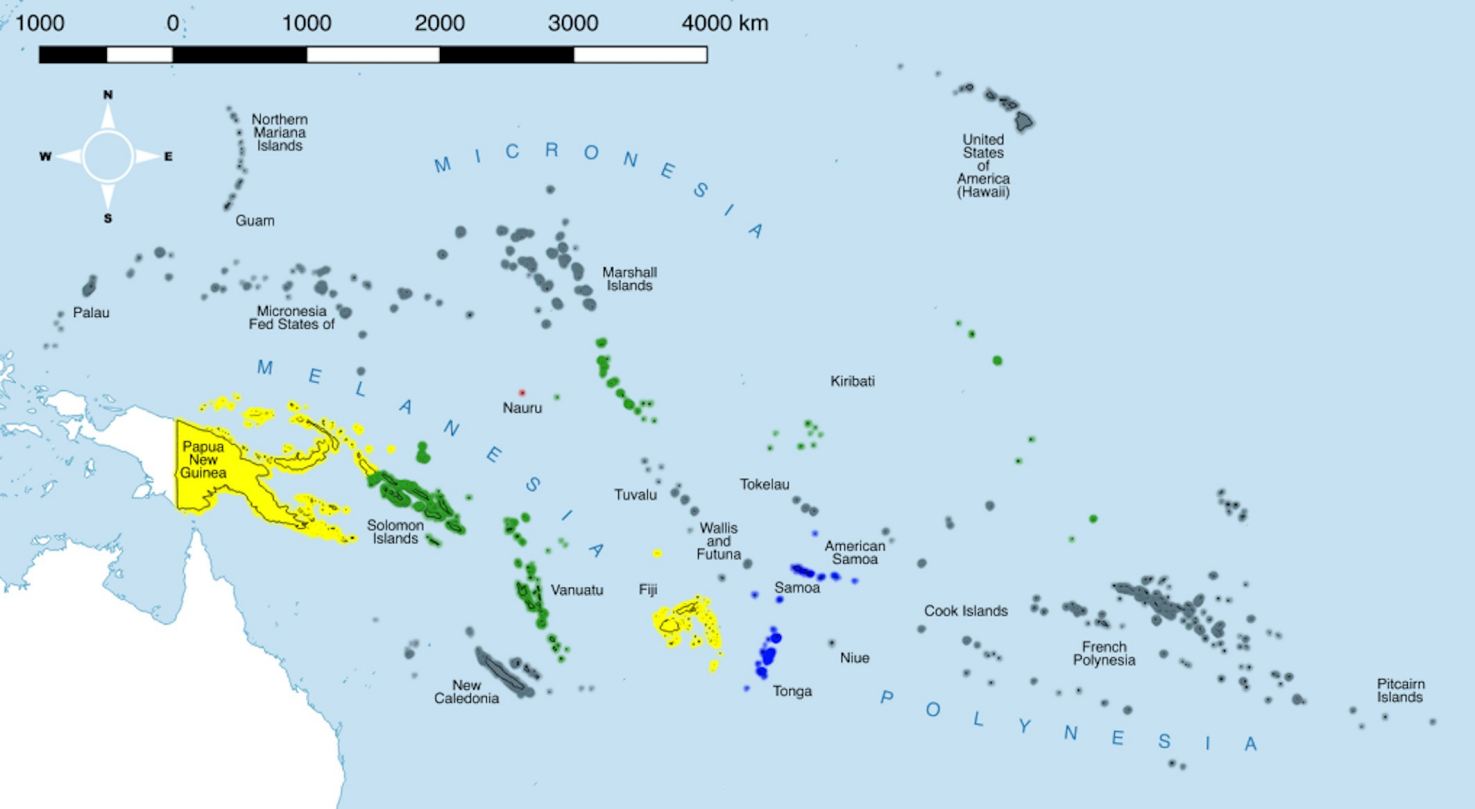
National-level summary of trachoma status following data extraction. Red: contemporary evidence of trachoma, current status unclear; yellow: some endemic districts, further mapping required, interventions not yet started; green: mapping complete, interventions implemented; blue: historic evidence of trachoma cases, no published contemporary data, current classification non-endemic; grey: no data or no reference to trachoma in available data or current classification non-endemic. Shapefiles downloaded from gadm.org and reproduced under a CC BY 4.0 license with permission from Dr Robert Hijmans. Map prepared in Miller projection using QGIS 2.16 [23].

The number of articles included in each stage of the systematic review process is described in Fig 1. From 1383 records identified with the initial search terms, 66 articles were eventually included in this review. Included articles were published between 1914 and 2018, with the period 2008–2018 having the highest frequency of included publications. An additional three papers, two doctoral theses, one report and one book section were found within the reference lists of the chosen full texts and were also included. The included articles are summarised in S3 Table. National-level outcomes are summarised in Fig 2.

### Quality assessment

The papers identified were a collection of laboratory studies, prevalence surveys, observational studies, conference abstracts and reviews. Of five reviews identified, three were specific to the Pacific [9,24,25] and two were reviews on trachoma or causes of visual impairment worldwide [26,27]. The primary publications relevant to the Pacific reviewed in these five articles were identified separately in this literature search. 35 articles specifically investigated trachoma. Prevalence estimates comparable to current mapping standards could only be derived from 8/66 studies (12.1%). Seven trachoma-specific population based prevalence surveys (PBSPs) were identified, five of which provided data from surveys related to the GTMP. A number of common issues precluded a number of studies generating prevalence estimates comparable to contemporary mapping standards. Five studies aimed to determine trachoma prevalence but only examined school attendance, thereby potentially missing trachoma cases where school attendance is low. Several papers either did not quantify the number of cases of trachoma, did not describe the specific clinical signs that were assessed, or did not provide sufficient description of methodology to indicate that trachoma was systematically assessed. Where trachoma data were presented as part of wider surveys surrounding visual impairment, the method of sample selection or data presentation were frequently inadequate to estimate prevalence. Many of the historical papers which specifically investigated trachoma failed to provide adequate detail on survey design, populations assessed and standardisation of grading, therefore, could not provide prevalence estimates comparable to recent mapping. Some historical studies did provide crude proportions, but did not use appropriate sampling methodology to extrapolate to district-wide prevalence with confidence.

### Melanesia

The majority of historical studies in this review focus on Papua New Guinea (PNG) and Fiji. One study from New Caledonia described cases of conjunctivitis from *Ct*, but did so in the context of urogenital strains infecting the eyes of neonates after birth [28]. Another extensive series of studies described the distribution of trachoma throughout Western New Guinea in 1964. This study revealed several areas where a high proportion of people had either clinical signs of trachoma, inclusion bodies, or both [29]. Western New Guinea is predominantly inhabited by people of Melanesian ethnicity, but is not within the scope of this review as it is part of Indonesia.

#### Papua New Guinea

Consecutive analyses of ophthalmic patient records (covering several regions between 1975 and 1991) consistently reported that trachoma was a minor contributor to visual impairment throughout PNG [30–32]. Exceptional areas where cases of entropion were found (for example, West Sepik and New Britain) were highlighted by the authors of these studies [32]. In their 1980s review of ophthalmic records in PNG, Parsons said “Trachoma is mentioned only to emphasise its relative unimportance in [PNG]” [32]. In outreach clinics which operated between 2011 and 2013, 35 cases of trachoma (clinical grade not specified) were identified from a single clinic. Despite identifying cases, no TT corrective surgeries were performed [33]. Surveys of visual impairment have also taken place in PNG, including a survey of over 3000 adults in Hanubada, Rigo, Rabaul and Teleformin regions (1979–1980). Participants were systematically and specifically examined for trachoma and other ocular morbidities. From that survey, Dethlefs asserted trachoma was found to be endemic in all areas studied, but was of “mild intensity and rarely caused visual deficit” [34]. More recently, a survey determining the causes of vision loss in older people living in the Port Moresby and Rigo coastal districts of PNG found cataracts and uncorrected refractive errors were the leading causes of visual impairment. Trachoma was not mentioned in this study [35]. During a Rapid Assessment of Avoidable Blindness in PNG, one case of trachomatous corneal opacity was found among 4818 survey participants aged ≥50 years from across the country, accounting for 0.8% of cases of blindness [36].

A number of surveys specifically focussed on trachoma have been conducted in PNG. Mann undertook extensive trachoma surveys throughout the country in the 1950s [37–40]. The surveys took place in the Marshall Bennett Islands, Trobriand Islands (Kitava and Woodlark), Samarai Island, Admirality Islands, Duke of York Islands, New Britain, New Ireland and Mainland PNG (The Highlands, Port Moresby, Sepik, Telefomin and Baiyer River). In total, over the 14 areas surveyed, 13,268 people were examined for clinical signs of trachoma using Mann’s own grading system [37] which was modified from MacCallan’s scheme [41]. The prevalence of trachoma (clinical grade not specified) ranged from 8.5% in the Marshall Bennett Islands to 85.8% in Teleformin. Although some of these estimates were extremely high, only 15 individuals suffered with bilateral blindness from trachoma. Mann stated that, in general, trachoma in PNG was “mild and self-healing”, with the exception of New Britain, where the blinding sequelae were common. Mann speculated that the geographic heterogeneity in prevalence was due to time since introduction of the disease to the different areas [40]. A survey in 30 accessible schools in Madang Province estimated 9.6% of 6153 surveyed children (mean age 10 years) to have moderate to severe trachoma (F3/F4 [F4 defined in this study as the total confluence of follicles on the tarsus] or P3) [42]. In 1971, a trachoma survey was conducted in a single village on one of the Admiralty Islands, all grades of trachoma according to the MacCallan grading scheme were rare [43]. This was in contrast to Mann’s survey data which found 64% of the 801 people examined on the Admirality Islands had trachoma (clinical signs not specified) [37].

A 2015 PBPS took place in six evaluation units (EUs) of PNG under the GTMP [16]. Overall 6555 children aged 1–9 years were examined alongside 8059 adults. Four of the EUs surveyed had TF prevalences of over 10%, these were Western Province, Southern Highlands (East), Southern Highlands (West) and West New Britain. Only Madang and National Capital districts had a prevalence of less than 10%. Across the entire survey, only four cases of TT were seen and these were all found in West New Britain, part of the only region where Mann found severe blinding trachoma [37]. The GTMP study also showed a strong association between unimproved sanitation and an increased risk of TF in children aged 1–9 years [16].

#### Fiji

A 1986 hospital-based study [44], found trachoma in 0.34% of the 800 patients (of all ages) surveyed. Surveys of blindness (outpatient questionnaires and hospital record screening) in Fiji rarely mention trachoma, with cataracts and refractive errors responsible for the majority of causes of visual impairment in both adults [45,46] and children [47]. Only two cases of trachoma (clinical grade not specified) were seen at outreach clinics 2011 and 2013, and neither underwent tarsal rotation surgery [33].

The earliest studies focussing specifically on trachoma were published in the 1930s [48,49]. These are both descriptive reports incorporating the authors’ clinical experiences with reports from medical colleagues working in-country. Neither presented any primary epidemiological data, although Stuppel published photographs of individuals with eye disease, at least one of which appeared to be a clear example of TT. Both studies concluded that trachoma was prevalent in Fiji [48,49]. Two studies which took place shortly after World War II examined trachoma in small groups of indigenous Fijians and also in expatriate American and New Zealand servicemen living in military facilities on Viti Levu. Transmission to visiting servicemen appeared, to these authors, to be nearly absent but clinical signs were seen in a high proportion of the Fijian study participants [50,51]. Prowasek-Halberstader inclusions were found in some cases with and without trachoma, and were associated with having trachoma [50].

The first estimates of trachoma prevalence in what might now be classed as a district- or sub-district-level population came from surveys in Viti Levu. These surveys used systematic grading systems or variations thereof such as Mann [37] Ching [52] to characterise trachoma. Swanston found 39% of almost 1400 people of all ages had clinical signs of trachoma including healed (scarred) trachoma [37,53]. Ward estimated a similar prevalence of trachoma at 35% in those under the age of 20 (although the author does not specify which grades), and almost 90% of those over the age of 60 (again the author does not specify the grade) [37,54]. Ward suggests in his report that the relatively high prevalence of trachoma in adults compared with the lower prevalences of active disease in children may indicate an improvement in living conditions in recent years resulting in a reduced incidence of active disease. Neither of these two studies demonstrate sufficiently random participant recruitment nor adjustment for age or gender to compare to modern prevalence estimates.

A trachoma rapid assessment (TRA) in 2007 demonstrated that active trachoma was present in the Western Division of Fiji [55]. No cases of trichiasis were found in 319 adults examined, although analysis of medical records demonstrated that surgical management of some TT cases had occurred in the three years preceding the survey [55]. Following this, a nation-wide survey was conducted in 2012 [10,15] and this reported estimates of TF prevalences exceeding 10% among 1 to 9 year-olds in all four Divisions of the country (mean: 15.4%, range: 10.4 [Suva]– 20.9% [Northern Division]). The survey also reported that in some regions active trachoma was more prevalent among people of the iTaukei ethnic group than among Indian Fijians but this was not a consistent across the Fijian divisions. Common household-level risk factors for trachoma, such as lack of availability of clean water or lack of facilites for disposal of human waste, were not found to be common in Fiji [10,15].

The Western and Northern Divisions of Fiji were shown to have very high TT prevalence (8.7% and 6.2%, respectively). However the authors treated this estimate with caution due to poor correlation with the relatively low TF estimates [15]. It was subsequently shown that a common sociocultural practice of eyelash epilation was prevalent in the Western Division of Fiji. A 2017 study [56] demonstrated that many Fijians who epilated (and who would have been classified as TT cases) in fact had no signs of scarring or entropion and were unlikely to be self-treating for the condition.

The Western Division was re-surveyed by PBPS in 2013 and conjunctival swabs were collected to assess the prevalence of ocular *Ct* infection with nucleic acid amplification tests (NAATs) [13]. This survey found ocular *Ct* to be rare (adjusted prevalence 2.3%), but, interestingly, also found TF and TT were much lower than the previous national survey had found (TF was only 2.9% in 1–9-year-olds compared with 19.6% seen in Western Province during the previous PBPS [10,15]). Serological data suggested that approximately 15% of 1-year-olds had been exposed to *Ct* at some point and that transmission of *Ct* between the ages of 1–15 years was likely to be very low [57]. The discrepancy between the two most recent population surveys of clinical trachoma is surprising and it is hard to understand how such a large difference could be explained by simple experimental variation, such as selection of clusters with differing TF prevalence by random chance. One key difference between the two studies was that the later survey utilised a standardised training scheme to ensure TF grading was as consistent as possible with surveys going on elsewhere in the world (i.e., GTMP compliance).

#### Solomon Islands

Subsequent to the initial report that suggested trachoma may be present in the Solomon Islands [49], Verlee and colleagues [58] examined trachoma in 600 people from inland villages in Bougainville (geographically part of the Solomon Island archipelago) and Malatia. Although the authors subsequently noted that trachoma was prevalent in other villages nearer the coast in those areas [59], they found no cases of trachoma in their initial survey. Serum samples and conjunctival scrapes taken from participants in the Solomon Islands, Indonesia, Australia and Tunisia were all used to test new culture methods for the isolation of *Ct* in the 1970s. *Ct* was isolated from a small number of Solomon Islander specimens collected from individuals with moderately severe active trachoma [60].

More recently, surgical outreach teams operating in the Solomon Islands carried out bilamellar tarsal rotation surgery on nine adults presenting for surgery with trachomabetween 2011 and 2013 [33]. Trachoma was confirmed as endemic when, in 2007, a TRA was conducted in nine of the 10 provinces of the Solomon Islands [55,61]. The published data from Malaita, Guadalcanal and Western provinces indicated that scarring was highly prevalent in adults living in the selected study villages, but did not identify a significant burden of TT, either during the survey, or in the prior analysis of hospital records. The scarcity of TT was such that the doctoral candidate who conducted the survey recommended infection testing in their thesis conclusion [62], and this was the subsequent topic of another doctoral thesis completed in 2017 [63]. In 2013, triggered by the TRA findings, PBPS took place in all provinces of the Solomon Islands. All provinces except Choiseul were found to have TF prevalence above 10% in the 1–9-year-old age group [10,12]. During the IAPB surveys in Malaita, Guadalcanal, Central Islands, Makira, Honiara City and Isabel provinces, data were collected on potential household risk factors for trachoma but association between these and TF were not formally evaluated. In Western, Choiseul, Temotu and Rennell and Bellona, household-level water, sanitation and hygiene data were also collected, but none of the variables measured were associated with increased risk of TF in the household. The TT prevalence in those aged >15 years in all EUs (Western, Choiseul, Temotu and Rennell and Bellona, Guadalcanal and Makira) was below 0.2% [10,12]. In Isabel province, the unadjusted TT prevalence was 0.3% [10].

The paucity of TT, despite the significant burden of TF, provided the impetus for a programme of research which questioned the aetiology of TF. This programme aimed to re-assess the burden of TF and test association with ocular infections, including direct quantitative NAATs for *Ct* infection. The prevalence of ocular *Ct* infection in children under 10 years-old in Temotu, Rennell and Bellona was 1.3%, and infection was significantly associated with TF. The authors considered this to be much lower than may have been expected in an area where TF was common [14]. A recent study found no association between severe scarring, classed using the FPC grading system [64,65], and age [66]. Seroepidemiological analyses also provided little evidence of ongoing intense transmission of infection [66,67]. Other pathogens (*Adenoviridae*, coagulase-negative *Staphylococcus*, *Haemophilus influenza*, *Moraxella catarrhalis*, *Staphylococcus pneumonia and Staphylococcus aureus*) were sought in these samples using PCR, but none appeared to have significant association with TF. There was also scant evidence for conjunctival dysbiosis, a characteristic disease-associated disturbance of the microbiological community [67]. A recent analysis of gene expression in the conjunctival epithelium of Solomon Islander *Ct*-negative TF cases provided no evidence of an allergic aetiology [68]. Whilst the causative factor of TF in these regions of the Solomon Islands remains unknown, it seems unlikely that the majority of clinical signs can be attributed to *Ct*, any other bacterial pathogen, or allergies; further work may be required to determine the aetiology of the disease.

#### Vanuatu

In 1989 a survey was carried out to determine the leading causes of blindness in the country. Cataracts was found to be responsible for 85% of binocular blindness and 67% of monocular blindness. Trachoma caused no binocular blindness but was found to be responsible for 5 cases (9%) of monocular blindness [69]. An evaluation of the first five years of a national eye care programme implemented in Vanuatu [70] also highlighted cataracts as the leading diagnosis from outpatient consultations, responsible for 25% of blindness. This study, however, found no cases of trachoma from the 29,109 records analysed. An outreach clinic survey showed that between 2011–2013, four cases of TT were seen, three of which underwent surgical treatment to correct the trichiasis [33].

In 2007 trachoma was studied in isolation during a TRA in Shefa and Tafea provinces [55]. Cases of TF, TI, TS and TT confirmed trachoma to be endemic, leading the Ministry of Health to utilise the GTMP platform to survey the entire country (excluding Luganville, Port Vila and Tafea province) in 2014. The adjusted prevalence of TF was 12%, and only two cases of TT were found in the ≥15-year-olds, an adjusted prevalence of 0.04%. TF was associated with living in a household with an unimproved latrine and larger family size. Older children in the 1–9 age group were also more likely to have TF than younger children in that age group [11].

### Micronesia

There are few studies on eye health in Micronesia. The US territories of Guam and the Northern Mariana Islands were not the subject of any of the articles included in this review.

#### Federated states of micronesia, palau and the marshall Islands

These countries are listed as non-endemic in the Global Health Observatory [20]. No publications that directly surveyed trachoma were found during this review. However, two general eye health surveys were identified. In 1987, a survey took place in Wotje Atoll of the Marshall Islands group. The project surveyed 400 indigenous adults and found the main causes of visual impairment to be uncorrected refractive error and cataracts. Cases of corneal opacity were reported in 24 people, many of which were bilateral. None of these cases were described as having the characteristic scarring patterns that are typical of trachoma [71]. Another study summarised data from 18 successive rounds of annual outreach clinics in the Federated States of Micronesia, Palau and Marshall Islands. Around 14,000 patients presented for assessment, from whom 2457 blind or visually impaired eyes were identified. Over half of these were due to cataracts, and the second most common cause (14% of eyes with low vision, 11% of blind eyes) was corneal opacity. The authors do not identify the aetiology of corneal opacity, but suggest that trachoma could be one of a number of key causes [72].

#### Kiribati

The first report of trachoma in Kiribati was from a field visit by Thylefors in 1989, who noted that 42% of an unspecified number of school children had evidence of TF and 4% had TI [73]. More recent data from a limited number of charitable outreach clinics in Kiribati indicated that there were no cases of trichiasis requiring surgery during 2013 [33]. An abstract presented at the 2014 Annual Congress of the Royal Australian and New Zealand College of Ophthalmologists did not mention trachoma as a cause of blindness in a random sample of 800 adults living in South Tarawa [74].

In 2007, a TRA was conducted in two districts of South Tarawa; cases of TF and TT were found [55]. A more detailed PBPS of South Tarawa and Betio [10] suggested that 22% of children aged 1–9 had TF and that of 959 people aged >15, around 1.5% had TT. Given the association between female gender and TT [75], this may be a slight overestimate as 94.4% of adult participants were female. Several environmental risk factors were observed as being prevalent among households that were surveyed in this study [10]. A second survey [76] was conducted in the major population hub of the Line Island group, Kiritimati Island. The age-adjusted prevalence of TF and TT was 29% and 0.2%.respectively. In those with TF, almost 50% had current ocular *Ct* infection. The prevalence of *Ct* infection throughout the surveyed child population was 27%. Seroreactivity to chlamydial antigen Pgp3 increased sharply between the ages of 1 and 6, and the majority of children were exposed by the age of 9, suggesting intense transmission of *Ct* among children in these communities.

#### Nauru

Nauru was included in the 2007 Pacific-wide TRA. Cases of TF were found in communities in four districts. However, very few cases of scarring and no cases of TT were found during the survey. Additionally, no cases of TT surgery were recorded at the Nauru Hospital over the four years preceding the survey [55].

### Polynesia

No published data were found on eyecare or trachoma in Tuvalu, French Polynesia, Wallis and Futuna or the Pitcairn Islands.

#### American Samoa

One early trachoma survey in American Samoa identified numerous cases of papillary trachomatous inflammation (MacCallan grade IIb). Harbert also indicated that the Department of Health in American Samoa had been investigating trachoma prior to that study [77]. In 1945, 422 inhabitants of American Samoa were surveyed to estimate trachoma prevalence. 217 participants showed signs of trachoma but neither the age or gender of the participants, nor the grading system that was used, were clear. Mann estimated 5% of blindness cases to be caused by trachoma, but it was subsequently suggested that this might be related to a social practice in which wooden picks are inserted beneath the eyelid to ‘alleviate irritation’. [37]. In 1975 a survey was designed to estimate trachoma prevalence in school-aged children from two islands (Tutuila and Ta’u) [78]. This involved examining 1030 school children (aged 3–19) from eight schools. The prevalence of active trachoma ranged from 9–42% and conjunctival scarring was seen in a number of children, however no trichiasis was seen. 154 adults were recruited from a local hospital, 12 of these with specific eye diseases and the remainder randomly selected in- or out-patients seen during the survey duration. Of these, 45 had evidence of past trachoma (e.g. pannus, conjunctival scarring or trichiasis) and four of these were blind as a result severe TT. Two of these individuals were seeking treatment at the time.

#### Cook Islands

A 1951 general survey of infectious diseases included conjunctivitis in the list of conditions that were studied. This survey was carried out in a single village on Rarotonga, the capital of the Cook Islands, but the study made no mention of trachoma [79]. Another survey that focussed on eye health was carried out in 1980 [80]. Two of the 986 adults surveyed had superior limbic pannus. The study concluded trachoma was not a major cause of blindness, although the investigators did not directly check the conjunctiva for follicles or scars.

There was no mention of trachoma found during a 2002 assessment of ophthalmic records from a single hospital in the Cook Islands, although the authors do note the large amount of missing data due to poor record keeping [81].

#### Hawaii

A 1956 study investigated the causes of blindness in Hawaii. Trachoma was not a cause of blindness in any of the 1677 people formally registered as blind [82].

#### Niue

Around 47% of the population on Niue were examined for trachoma in 1945 [37]. 574/1867 were reported to be affected by trachoma but the age, gender and clinical disease profile of these individuals was not provided. Disease was reported as unevenly spatially distributed.

#### Samoa

The earliest report found during the literature search was a 1914 investigation into epidemic eye disease in Samoa [83]. Leber and Prowazek noted that trachoma was present in Samoa alongside other eye diseases. [83–85].

A small survey in 1945 found 135/245 of people had trachoma, although the criteria for participation, the clinical grade, the gender and age of those affected was not described [37]. A 1958 study examining over 700 adult ophthalmic outpatients and inpatients found 15 to have active trachoma and 24 to have visible trichiasis. Trachoma was deemed to be responsible for 10/136 cases of blindness [86]. In 1982, a survey of 510 patients found no cases of active trachoma but 19 patients with cicatricial entropions of the upper lids due to trachoma. A number of these patients had significant corneal scars and entropion surgery was performed [87]. Samples from Samoans of undefined age but with active trachoma were used in a thorough evaluation of laboratory methods for identifying ocular *Ct* infection. Of 20 specimens, 16 (80%) were seroreactive by microimmunefluorescence testing, but *Ct* could not be cultured in McCoy cells from any of these participants. Of those who were seropositive, 11 reacted strongly to serovar A antigens [60].

No record of trachoma was identified during a 2002 screening of 380 adult ophthalmic patient records from a single hospital in Samoa. Cataracts and refractive errors were cited as the major causes of visual impairment and blindness [45]. The most recent study focusing on eye health in Samoa was a review of clinical records from charitable outreach clinics between 2011–2013; no cases of trachoma were documented [33].

#### Tokelau

In 1963, a general eye health survey was conducted throughout Tokelau. 98% of 1862 inhabitants were surveyed, and only one had follicles and pannus. 14 people had signs of severe or scarred trachoma, although these were suspected to be immigrant cases from Samoa [88].

#### Tonga

A 1991 survey of binocular blindness in people living in randomly selected villages in Tonga concluded that three out of 4056 participants aged >20 years were blind in both eyes as a result of trachoma [89], although no further information was given on the method for evaluation and management of the cases that were found. No cases of trachoma were identified during two studies of patient records, one from a single clinic in Tonga (2002) [45] and the other from charitable outreach services (between 2011–2013) [33].

## Discussion

A key question this study set out to address was what, if any, further mapping is needed to complete the baseline map of trachoma in the Pacific region. We found there to be only seven studies suitable for providing trachoma prevalence estimates. This highlights the paucity of data available to policy makers as they decide how best to manage trachoma in the Pacific region. This review has demonstrated the value of standardised data collection when making comparisons between surveys. Therefore, in those countries where further maping is required, we feel that mapping should adhere to standardised trachoma mapping strategies (TRA/PBPS). In addition, given the findings from the Solomon Islands, infection testing should become routine for trachoma mapping in the Pacific, and should also have a role in trachoma mapping elsewhere in the world. TRA-type targeted studies are not suitable for serological assessment given the small sample size and non-population-based sampling method, but NAAT testing for ocular *Ct* infection could be useful to determine the association between inflammation and infection. For PBPS-type district-level surveys, serological testing alongside NAATs has been a powerful tool in the mapping undertaken so far and should be incorporated as standard into future mapping efforts in the Pacific.

Solomon Islands, Kiribati and Vanuatu have the clearest picture of trachoma endemicity following recent, nationwide baseline mapping. In Kiribati, trachoma is clearly a public health problem, and further mapping can be postponed until SAFE interventions have had a chance to impact on trachoma prevalence. In the Solomon Islands and Vanuatu, the apparent paucity of TT and ocular *Ct* infection requires further investigation, particularly of the potential that TF has a causative origin that does not include bacterial infections. The data from recent studies of aetiology justifies a different approach to that utilised in Kiribati and other sub-Saharan African countries with similar TF prevalence. This could include cessation of MDA after a single round in districts with a TF prevalence of >10% in those aged 1–9 years. However, given the predictions of rapid population growth and potential for overcrowding in urban areas of Solomon Islands and Vanuatu [90], the F and E components of the SAFE strategy could still be justified alongside stringent surveillance to ensure the rate of ocular *Ct* transmission does not increase with the increase in population density.

The epidemiology of trachoma in PNG is similar to that of its Melanesian neighbours in the districts mapped so far, suggesting that it too may not require repeated rounds of MDA to reduce prevalence of *Ct* infection. The outcome of ongoing research activities in PNG, Solomon Islands and Vanuatu will be useful to guide mapping and treatment requirements. Review of ophthalmic records or deployment of the TRA protocol may be warranted in areas where trachoma has historically been described as more severe, such as East New Britain and West Sepik, to determine whether that remains the case. Infection testing should be incorporated to ensure the data is comparable to other areas in the Pacific. In Fiji, there is a need to further investigate the discrepancy between the two recent pre-MDA surveys in the Western Division; the outcome of those surveys and the other work in Melanesia will inform the subsequent intervention strategy. Where futher mapping is conducted, inclusion of infection testing (either through NAATs at the individual level or serology at the population level) could serve to further identify areas for targeted control. There is evidence of TF in Nauru, so population-based mapping is justified, again serological monitoring and infection testing may be useful here.

In Samoa, cases of active and scarring trachoma seem to have been identified in previous surveys, but no recent systematic data have been published. No eye-health data were identified in Tuvalu and very little from Tonga. These three independent Pacific Island states may face similar socioeconomic challenges to the independent Melanesian countries in terms of infrastructure and access to healthcare in rural areas, and may therefore be at risk of trachoma endemicity. As such, we believe use of the TRA protocol combined with NAAT infection testing is warranted unless the respective governing bodies of these countries have national disease surveillance data suggesting otherwise.

With the exception of American Samoa, where historic reports indicate trachoma has been a public health problem, no historical data were available from the majority of territories with economic links to developed nations included in this review (New Caledonia, Guam, Northern Mariana Islands, Pitcairn Islands, Wallis and Futuna and French Polynesia). The relationship between these states and developed nations mean that residents can move freely between them, and they enjoy greater economic stability, infrastructure and access to higher standards of healthcare. We therefore do not expect many risk factors for trachoma to be prevalent. Similarly, those in free association with New Zealand (Tokelau, Niue and Cook Islands) or the United States (Palau, Marshall Islands and Federated States of Micronesia) are also less likely to experience risk factors for trachoma. A handful of cases have been described in historic surveys of the causes of blindness; these are a tiny proportion of the whole and are poorly characterised, so we cannot be certain they meet definitions of trachoma by contemporary standards. If trachoma has been detected or is detected in the future during ongoing routine and targeted surveillance by respective governmental health services, a TRA could be a useful tool to assess whether more detailed mapping is required in these populations.

The second objective of this review was to identify whether any evidence could be found that may describe the temporal patterns of trachoma in this region over the past century. While good contemporary data on trachoma from recent population-based surveys is available, such robust evidence was almost absent amongst historical reports, with four key exceptions. The Pacific-wide mapping by Mann in the 1940s and 1950s [37], Ward’s surveys of Fiji [54], Dethlefs’ various surveys of trachoma in PNG [34] and Ostler’s and Thygeson’s surveys in American Samoa [78] stood out for their systematic nature and large scale. However these studies shared critical limitations as they did not differentiate between the various clinical signs of trachoma that were observed, nor were the participant selection procedures suitably random to allow extrapolation of prevalence estimates to areas outside of the specific islands and villages studied.

One key conclusion from the aggregated data is that ophthalmologists have consistently identified cases of the late stages of trachoma throughout the region between 1914 and 2018, but none have considered it to be worthy of special mention in general eye-health reports, nor have there been calls for or implementation of major strategies for clinical management of TT. Where it has been available by clinical outreach or in the routine clinical setting, uptake of TT surgery has been consistently low throughout the Pacific. Interestingly, the discrepancy observed between TF and TT in contemporary data has been alluded to a number of times in surveys in PNG [34]. From the contemporary mapping data available, countries with a predominantly Melanesian population seem to have low TT prevalence may be an artefact of the intensive mapping in these countries compared to elsewhere in the Pacific. However, that this has coincided with a low *Ct* prevalence may be an artefact of their being the most accurately mapped, or may lead some to propose that there are protective cultural or genetic factors related to Melanesian heritage. While such correlations can lead to over-interpretation, research into this phenomenon is ongoing.

There are limitations to this study. Firstly, the search method used did not include systematic assessment of grey literature databases nor request unpublished information from local stakeholders (for example, Ministries of Health and non-governmental organisations working in the region). If these search tools had been utilised, it is possible there would have been more studies to include in the review including information indicating data from countries that have been classed as non-endemic by the WHO. However, we wanted to ensure our findings were based on public information sources that could be independently verified. Secondly, although most of the articles retrieved by the literature search were accessible there were two conference abstracts that could not be accessed. Finally, some of the primary studies described in Mann’s book “Culture, Race, Climate and eye disease” were not accessible, so secondary data presented by Mann has been presented here.

This review has highlighted the heterogeneous pattern of trachoma throughout the Pacific. By summarising various studies and survey results into a single document, we hope to provide a resource for policymakers to identify key priority areas for mapping and further investigation in the peri-elimination period.

## Supporting information

S1 Table PRISMA checklist required by journal publication guidelines. (DOC)

S2 Table Quality control questions used in the evaluation of trachoma studies. (DOCX)

S3 Table A summary of all the articles included in this review. This table includes the quality control assessment used on the prevalence studies.
(DOC)
