# Supplementary material for: A systematic review of historical and contemporary evidence of trachoma endemicity in the Pacific Islands
Source: PLoS One. 2018 Nov 15;13(11):e0207393. doi: 10.1371/journal.pone.0207393 (PMC6237375; doi:10.1371/journal.pone.0207393)
Supplement: S2 Table — (DOCX) [file pone.0207393.s002.docx]

**Supplementary table 1: Quality control questions used in the evaluation of trachoma studies**

| **Quality control question number** | **Quality control questions (adapted from Munn [22])** |
| --- | --- |
| **1** | Was the sample representative of the district or sub-district level population? |
| **2** | Were the study participants recruited in a suitably random way? |
| **3** | Was the sample size adequate? |
| **4** | Were the study subjects and the setting described in detail? |
| **5** | Were standardised trachoma grading methods used? |
| **6** | Was the condition measured reliably*? |
| **7** | Was there appropriate statistical analysis? |
| **8** | Are all important confounding factors/subgroups/differences identified and accounted for**? |
| **9** | Was the response rate adequate***, and if not, was the low response rate managed appropriately? |
| * In the context of this review, we interpret this to mean description of training, certification and ongoing monitoring of graders.  ** In the context of this review, we consider age and gender to be the key potential confounders for prevalence estimates.  *** We set and arbitrary threshold of >70% for this review. | |
