# Supplementary material for: A systematic review of historical and contemporary evidence of trachoma endemicity in the Pacific Islands
Source: PLoS One. 2018 Nov 15;13(11):e0207393. doi: 10.1371/journal.pone.0207393 (PMC6237375; doi:10.1371/journal.pone.0207393)
Supplement: S3 Table — This table includes the quality control assessment used on the prevalence studies. (DOC) [file pone.0207393.s003.doc]

**S3 Table: A summary of all the articles included in this review.** This table includes the quality control assessment used on the prevalence studies.

| **Study details** | | | | | **Quality Control Criteria * (see supplementary table 1 for the list of quality control questions)** | | | | | | | | | |
| --- | --- | --- | --- | --- | --- | --- | --- | --- | --- | --- | --- | --- | --- | --- |
| **Lead author, study year if known [reference]** | **Country, District** | **Study focus** | **Grading scheme** | **Laboratory methods** | **1** | **2** | **3** | **4** | **5** | **6** | **7** | **8** | **9** | **Notes** |
| Lee [36] | PNG | Rapid assessment to determine prevelance of visual impairment and blindness in adults ≥50 | Not specified | NA | Yes | Yes | Yes | Yes | No | No | Yes | No | Yes | Only older adults examined and no specific trachoma examination |
| Vasileva, September 2013 [68] | Solomon Islands, Temotu and Rennell & Bellona | A study aiming to charecterise patterns of gene expression in children with and without TF and/or Ct infection. | WHO Simplified grading scheme | Host transcriptomics and ddPCR for *Ct* DNA detection from conjunctival swabs | NA | NA | NA | NA | NA | NA | NA | NA | NA | - |
| Butcher, September 2013 [67] | Solomon Islands, Temotu and Rennell & Bellona. | A study to determine if active trachoma was associated with a common non-chlamydial infection or with a dominant polymicrobial community dysbiosis. | WHO Simplified grading scheme | 16S rRNA amplicon sequencing for microbiome analysis and ddPCR for pathogen detection | NA | NA | NA | NA | NA | NA | NA | NA | NA | - |
| Butcher, June 2015 [66] | Solomon Islands,  Temotu and Rennell & Bellona. | Use of age-specific profiles of Ct antibodies and conjunctival scarring to determine whether there is evidence of ongoing transmission and pathology from ocular *Chlamydia trachomatis (Ct)*infection. | FPC for scarring severity of conjunctival photos | Ocular swabs for detection of *Ct* DNA by ddPCR, capillary blood for anti-Pgp3 antibody analysis | NA | NA | NA | NA | NA | NA | NA | NA | NA | - |
| Cama,  November 2015 [76] | Kiribati, Kiritimati Island | Cross-sectional GTMP survey to determine the prevalence of active trachoma and TT in Kiritimati. | WHO simplified grading scheme | Ocular swabs for detection of *Ct* DNA by ddPCR, capillary blood for anti-Pgp3 antibody analysis | Yes | Yes | Yes | Yes | Yes | Yes | Yes | No | Yes | Clustering wasn't accounted for when generating confidence intervals |
| Taleo,  October–November 2014 [11] | Vanuatu, Torba, Penama, Shefa, Malampa and Sanma | Cross-sectional GTMP survey to generate prevalence estimates of TF, TI and TT. | WHO simplified grading scheme | NA | Yes | Yes | Yes | Yes | Yes | Yes | Yes | Yes | Yes |  |
| Butcher [63] | Solomon Islands, Fiji and | Doctoral thesis | NA | NA | NA | NA | NA | NA | NA | NA | NA | NA | NA | Thesis so non-peer reviewed studies |
| Macleod,  November–December 2013 [56] | Fiji,  Western Division | Used a questionnaire to determine if the common practice of eyelash epilation was to self-treat TT. Examined all adults admitting to epilation for signs of TS and TT. | WHO simplified grading scheme | NA | NA | NA | NA | NA | NA | NA | NA | NA | NA | - |
| Cocks,  July–August 2015 [57] | Fiji,  Western Division | Cross-sectional survey to determine prevalence of anti-Pgp3 antibodies in children aged 1-14 years. | NA | Capillary blood for anti-Pgp3 antibody analysis | NA | NA | NA | NA | NA | NA | NA | NA | NA |  |
| Butcher,  September–November 2013 [14] | Solomon Islands,  Temotu and Rennell & Bellona | Cross-sectional survey to estimate the prevalence of trachoma and ocular *Ct* infection. | WHO simplified grading scheme | Ocular swabs for detection of *Ct* DNA by ddPCR. | Yes | Yes | Yes | Yes | Yes | Yes | Yes | Yes | Yes |  |
| Macleod,  November–December 2013 [13] | Fiji,  Western Division | Cross-sectional GTMP survey to determine prevalence of active trachoma and *Ct* infection | WHO simplified grading scheme | Ocular swabs for detection of *Ct* DNA by ddPCR. | Yes | Yes | Yes | Yes | Yes | Yes | Yes | No | Yes | High number of individuals per household. |
| Ko,  October–December 2015 [16] | PNG,  Morobe, Southern Highlands, West New Britain, Madang, Western, National Capital District. | Cross-sectional GTMP survey to determine TF, TI and TT prevalence | WHO simplified grading scheme | NA | Yes | Yes | Yes | Yes | Yes | Yes | Yes | Yes | Yes |  |
| Sokana,  September–November 2013 [12] | Solomon Islands,  Choiseul, Western, Rennell & Bellona and Temotu | Cross-sectional GTMP survey aimed to estimate the baseline prevalence of trachoma. | WHO simplified grading scheme | NA | Yes | Yes | Yes | Yes | Yes | Yes | Yes | Yes | Yes |  |
| Lees,  2011–2013 [33] | Fiji; Kiribati; Samoa; PNG; Solomon Islands; Tonga;Vanuatu | A review of data from outreach eye health clinics to establish the primary reasons for people seeking eye care | NA | NA | NA | NA | NA | NA | NA | NA | NA | NA | NA |  |
| Tekeraoi [74] | Kiribati, Tarawa | Population-based, cross-sectional study to determine main causes of visual impairment in adults >45 years in living in urban Kiribati | Not specified | NA | Yes | Yes | UC | No | No | No | Yes | UC | UC | Only older adults exained and No specific trachoma examination |
| Keeffe [9] | Fiji; PNG; Tonga; Vanuatu | A systematic review from 1990 to 2010 about the leading causes of visual impairment and blindness in SE Asia and Oceania. | NA | NA | NA | NA | NA | NA | NA | NA | NA | NA | NA |  |
| Kama, 2012 [15] | Fiji,  Central, Eastern, Western and Northern Division, Suva | Cross-sectional population prevalence survey to produce baseline trachoma prevalence estimates for Fiji | WHO simplified grading scheme | NA | Yes | Yes | Yes | Yes | Yes | Yes | Yes | Yes | Yes |  |
| Kline [24] | NA | A review of the NTDs affecting people living in Oceania | NA | NA | NA | NA | NA | NA | NA | NA | NA | NA | NA |  |
| Pikacha, 2007 [61] | Solomon Islands | Conference abstract presenting data from Pacific-wide TRA | WHO simplified grading scheme | NA | No | No | UC | Yes | Yes | UC | No | UC | UC | Conference abstract – methodology not explained and not all data presented |
| Ramke, Septmber–November 2009 [46] | Fiji, Viti Levu | A survey following the (slightly modified) rapid assessment for blindness methodology to determine the causes and prevalence of visual impairment and vision loss in adults >40 years in Fiji. | Not specified | NA | Yes | Yes | Yes | Yes | No | No | Yes | Yes | Yes | Only older adults examined and no specific trachoma examination |
| International Agency for the Prevention of Blindness,  2012  [10] | Kiribati, South Tarawa and Betio;  Fiji, Northern, Central, Eastern and Western Disvisions and Suva; Solomon Islands**,** Guadacanal, Makira, Isabel, Malatia and Honiara | Cross-sectional survey to determine prevalence of active trachoma and potentially blinding trachoma | WHO simplified grading scheme | NA | Yes | Yes | Yes | Yes | Yes | UC | Yes | no | UC | Trachoma grading certification not mentioned. Much higher proportion of women sampled and response rate not clear. |
| Mathew [62] | Fiji, Kiribati, Vanuatu, Solomon Islands and Nauru | Doctoral thesis | NA | NA | NA | NA | NA | NA | NA | NA | NA | NA | NA |  |
| Cama [47] | Fiji, Viti Levu | An analysis of data from informal registers for the visually impaired, school vision screening programs, key informants and cases from a hospital, to establish prevalence and causes of visual impairments in Fijian children <15 years | NA | NA | Yes | No | Yes | Yes | No | No | Yes | Yes | UC | Participants not recruited randomly, not a specific trachoma examination, response rate not clear. |
| Mathew,  2007 [55] | Kiribati, Betio and Buota; Nauru, Aiwo, Boe, Location district and 1 unnamed district;  Vanuatu,  Shefa and Tafea;  Fiji, Western division;  Solomon Islands, Malaita, Western and Guadalcanal | Trachoma rapid assessment to determine if trachoma is endemic | WHO simplified grading scheme | NA | No | No | No | Yes | Yes | Yes | No | No | UC | Chose to sample “high risk” communities, actively sought those with TT, small sample size. Didn’t aim to generate prevalence estimates |
| Williams, 2006 [70] | Vanuatu | A review of a national eye health programme, achieved by analysing clinic data records from annual surgical logs, outreach and clinic reports and patient records from 2000-2005 | NA | NA | NA | NA | NA | NA | NA | NA | NA | NA | NA |  |
| Ramke,  2002 [45] | Cook Islands, Rarotonga; Fiji, Suva; Samoa, Upolu; Tonga,  Tongatopu | A hospital-based rapid assessment involving random selection and analysis of patient records to determine the main causes of visual impairment and blindness. | NA | NA | NA | NA | NA | NA | NA | NA | NA | NA | NA |  |
| Garap  December 2004–March 2005 [36] | PNG, Port Moresby and Rigo Coastal District | Used the WHO Rapid Assessment of Cataract Surgical Services protocol to determine the prevalences and causes of visual impairment and blindness in Papua New Guinea | NA | NA | Yes | Yes | Yes | Yes | No | No | Yes | Yes | Yes | No specific trachoma examination |
| Polack [26] | Global | Global mapping of trachoma summary of 18 years of data collection consisting of 139 PBPS from 33 countries | NA | NA | NA | NA | NA | NA | NA | NA | NA | NA | Na |  |
| Pascolini [27] | 68 countries including Tonga and Vanuatu | A literature review which compiled all available epidemiological data on the causes and prevalence of blindness and visual impairment globally. | NA | NA | NA | NA | NA | NA | NA | NA | NA | NA | NA |  |
| Yamamoto [72] | Palau, Federated States of Micronesia and the Marshall Islands | A retrospective study looking at the causes of blindness in Micronesian islands over the course of 18 years seen by a team of ophthalmologists and nurses visiting annually. All cases were reviewed. | Not specified | NA | NA | NA | NA | NA | NA | NA | NA | NA | NA |  |
| Keeffe [25] | Region-wide | A general review of the causes of vision loss in the pacific region. | NA | NA | NA | NA | NA | NA | NA | NA | NA | NA | NA |  |
| Newland, 1991 [89] | Tonga | Used stratified cluster sampling to identify causes of blindness in adults > 20 years in Tonga | Not specified | NA | Yes | Yes | Yes | Yes | No | No | Yes | Yes | Yes | No specific trachoma examination |
| Negrel, 1989 [73] | Kiribati | An analysis of the use of the new simplified grading system involving a school visit in Kiribati | WHO simplified grading scheme | NA | NA | NA | NA | NA | NA | NA | NA | NA | NA |  |
| Newland,  March–April 1989 [69] | Vanuatu | A cluster-randomised survey to determine causes and prevalence of blindness | Not specified | NA | Yes | Yes | Yes | Yes | No | No | Yes | No | UC | No specific trachoma examination, response rate not mentioned |
| Parsons, 1980–1989 [32] | PNG, Madang | A review of quarterly reports from an ophthalmic practice in Madang over the previous ten years to determine the primary reasons for people seeking eye care. | NA | NA | NA | NA | NA | NA | NA | NA | NA | NA | NA |  |
| Wessels,  May 1987 [71] | Marshall Islands | A survey to determine general levels of eye health in one of the Marshall Islands | Not specified | NA | No | Yes | Yes | Yes | No | No | No | No | No | No grading scheme or evidence of grader training. Sample population not representative. |
| Parsons,  January 1980- December 1984 [30] | Papua New Guinea  Madang Province and other coastal and highland areas | An analysis of available out-patient records from a 5 year period to determine the causes of blindness and visual impairment in Papua new Guinea | NA | NA | NA | NA | NA | NA | NA | NA | NA | NA | NA |  |
| Andrist July and August 1983 [44] | Fiji | Determining the ocular diseases causing significant harm in Fiji by examination of people attending eye-clinics | Not specified | NA | NA | NA | NA | NA | NA | NA | NA | NA | NA |  |
| Egbert  1982 [87] | Western Samoa  National hospital of Apia | A hospital based survey to determine the causes of blindness in 510 eye-patients | NA | NA | NA | NA | NA | NA | NA | NA | NA | NA | NA |  |
| Heriot  September 1980 [80] | Cook islands  Rarotonga Islands | A survey of 986 inhabitants of the Cook islands to discover if there were eye health complications as a result of diabetes and carry out a general eye-health survey | Trachoma wasn't graded and eyelids weren’t everted. | NA | No | Yes | Yes | Yes | No | No | No | No | Yes | No grading scheme or evidence of grader training. Sample population not representative. |
| Dethlefs,  1979–1980 [34] | PNG, Hanuabada-Port Moresby, Rigo district of central province. Rabaul and Telefomin | To establish trachoma prevalence in certain districts of PNG using a mixture of sampling methods | Maccallan | NA | No | No | UC | Yes | Yes | No | No | No | UC | Participants always randomly recruited, no information on grader training. |
| Parsons [42] | PNG,  Madang | Examined as many children as possible from 30 of the 121 community schools in the Madang district to determine rates of trachoma in these children. | FPC | NA | No | No | Yes | Yes | Yes | No | No | No | UC | Only school children sampled |
| Kluxen 1910-1913 [85] | Samoa; Mariana Islands | A summary and review of investigations of the epidemic eye diseases found in Samoa, that took place in the early twentieth century | NA | NA | NA | NA | NA | NA | NA | NA | NA | NA | NA |  |
| Kluxen 1910-1913 [84] | Samoa | A summary and review of investigations of the epidemic eye diseases found in Samoa, that took place in the early 20th century | NA | NA | NA | NA | NA | NA | NA | NA | NA | NA | NA |  |
| Parsons  January 1971–December 1973 [30] | PNG,  New Guinea Mainland | An analysis of patient records from a 3-year period to establish which ophthalmic services were available in a single hospital in PNG at the time. | NA | NA | NA | NA | NA | NA | NA | NA | NA | NA | NA |  |
| Damon [59] | Solomon Islands | An biomedical-anthropological study examining the causes of different ailments amongst different Solomon Islands tribal groups | NA | NA | No | No | Yes | Yes | No | No | No | No | Yes |  |
| Jones [60] | Western Samoa; Solomon Islands | Examined different laboratory methods of Chlamydia trachomatis diagnosis and involves the use of samples from Solomon Islands and Samoa. | Not specified | Culture in chick embryos and McCoy cells, MIF | NA | NA | NA | NA | NA | NA | NA | NA | NA |  |
| Heath, 1971 [43] | PNG,  Peri, Great Admiralty Islands | A combined anthropological study and an ophthalmic survey of all inhabitants from a single village. | Maccallan’s classification of trachoma | NA | NA | NA | NA | NA | NA | NA | NA | NA | NA |  |
| Ostler [78] | American Samoa  Tutila & Ta'u Islands | Survey to estimate trachoma prevalence in American Samoa involving random selection of school attending children, teachers and adults attending hospitals/clinics | Diagnosis based on presence of 2 clinical signs (follicles, conjunctival scarring, Herbert’s Pits or TT | Conjunctival scrapings and blood samples taken from children with active trachoma | No | No | Yes | Yes | Yes | No | No | No | UC | Mostly school attending children examined, no information on trainer grading. |
| Verlee [58] | Solomon Islands,  Bougainville, Malaita | An ophthalmic survey of two tribal groups in the Solomon Islands, aiming to sample everyone in the chosen villages | Not specified | NA | No | No | Yes | Yes | No | No | No | No | UC |  |
| Mann, 1945 [37] | PNG | Trachoma prevalence survey across PNG | Maccallan and Mann | NA | No | No | Yes | Yes | Yes | UC | No | No | UC |  |
| Mann, 1945 [37] | American Samoa; Western Samoa; Niue | To determine the endemicity status and prevalence of trachoma | Not specified | NA | No | No | Yes | No | Yes | UC | No | No | UC | Secondary data taken from earlier surveys |
| Mann,  1945 & 1955 [37] | Fiji, Viti Levu | Reporting the results of previous trachoma surveys in Fiji | Not specified | NA | No | No | Yes | No | Yes | UC | No | No | UC | Secondary data taken from earlier surveys |
| Mann, 1954 [40] | PNG | To examine the variability of trachoma and how and when it may have arrived in PNG | Not specified | NA | NA | NA | NA | NA | NA | NA | NA | NA | NA | No primary data - this is a reinterpretation of data from Mann book |
| Ward, 1955 [54] | Fiji, Viti Levu | A large-scale study on trachoma prevalence in Fiji taking place in villages said to be representative of the various climatic, social and geographical conditions of Viti Levu | Ching's and Mann's | NA | Yes | UC | Yes | Yes | Yes | No | No | No | UC |  |
| Elliot,  July 1963 [88] | Tokelau,  Anafi, Nukunonu, Fakaofo | To determine which eye diseases are found on three islands of Tokelau by examining all available inhabitants | Not specified | NA | Yes | Yes | Yes | Yes | No | No | No | No | Yes |  |
| Elliot, 1959 [86] | Samoa | To diagnose conditions of 700 eye-hospital outpatients to determine causes of eye disease in Samoa. Recruitment took place at Apia General Hospital | Not specified | NA | NA | NA | NA | NA | NA | NA | NA | NA | NA |  |
| Mann [38] | PNG | To determine when trachoma first arrived in Australasia | Maccallan and Mann | NA | NA | NA | NA | NA | NA | NA | NA | NA | NA | No primary data - this is a reinterpretation of data from Mann book |
| Mann  [39] | PNG | To determine if there are cultural or ethnic correlates with disease incidence or severity | Maccallan and Mann | NA | NA | NA | NA | NA | NA | NA | NA | NA | NA | No primary data - this is a reinterpretation of data from Mann book |
| Pinkerton  [82] | Hawaii | To determine the causes of blindness of those registered blind in Hawaii | NA | NA | NA | NA | NA | NA | NA | NA | NA | NA | NA |  |
| Swanston,  February–April and June–July 1945 [53] | Fiji, Viti Levu | A survey to determine trachoma prevalence in Fiji | Not specified | NA | UC | UC | UC | Yes | No | No | No | No | No |  |
| Faine,  January–February 1950 [79] | Cook Islands,  Rarotonga | A survey to determine which infectious diseases were endemic amongst the inhabitants of Rarotonga, Cook islands | Not specified | NA | No | UC | UC | No | Yes | No | No | No | No |  |
| Bodian [50] | Fiji | A study using new diagnostic tools to determine if indigenous Fijians, and American soldiers had signs of clinical disease and positive results using a new conjunctival swab staining technique. | Not specified | Powazek-Halberstadter inclusion bodies | NA | NA | NA | NA | NA | NA | NA | NA | NA |  |
| Hope-Robertson [51] | Fiji | A study of New Zealand army personnel based in Fiji to determine if they were affected by trachoma. | Not specified | NA | NA | NA | NA | NA | NA | NA | NA | NA | NA |  |
| Harbert [77] | American Samoa | A descriptive study on the trachoma and eye disease in American Samoa | Maccallan | NA | NA | NA | NA | NA | NA | NA | NA | NA | NA |  |
| Maccallen [49] | Fiji; Solomon Islands | To describe trachoma in the British colonial empire including correspondence with medical officers in Fiji and Solomon Islands | Not specified | NA | NA | NA | NA | NA | NA | NA | NA | NA | NA |  |
| Stuppel [48] | Fiji | A descriptive study of trachoma in Fiji | Not specified | NA | NA | NA | NA | NA | NA | NA | NA | NA | NA |  |
| Leber [83] | Samoa | A descriptive study of the epidemic eye diseases seen in Samoa | Not specified | NA | NA | NA | NA | NA | NA | NA | NA | NA | NA |  |
| *Quality control criteria only assessed in studies that set out to estimate prevalence of trachoma and/or eye disease.  Ct: *Chlamydia trachomatis*; ddPCR: droplet digital PCR; NTD: Neglected Tropical Disease; PNG: Papua New Guinea; GTMP: Global Trachoma Mapping Project; TRA: Trachoma Rapid Assessment; UC: Unclear, | | | | | | | | | | | | | | |
